# Production of low‐protein cocoa powder with enzyme‐assisted hydrolysis

**DOI:** 10.1002/fsn3.3997

**Published:** 2024-01-29

**Authors:** İnci Cerit, Könül Mehdizade, Ayşe Avcı, Omca Demirkol

**Affiliations:** ^1^ Department of Food Engineering Sakarya University Esentepe Sakarya Turkey

**Keywords:** amino acid disorder, cocoa powder, enzyme‐assisted hydrolysis, low‐protein product

## Abstract

Amino acid‐related disorders are caused by a defect in the metabolic pathways of amino acid groups. These patients must follow a lifelong protein diet. The objective of this study was to produce a low‐protein cocoa powder (LPP) with enzymatic hydrolysis and precipitation method. First, the solubility of cocoa powder was increased by heat and enzyme treatments (Amylase, Viscozyme, and Alcalase). Then, the protein level was decreased by isoelectric precipitation. According to the obtained results, the solubility of cocoa powder rose from 28.61% to 50.69%. Protein content decreased by almost 40% and significant reductions in the amino acid profile were also provided; the highest ones were detected in methionine (100%), lysine (73.65%), leucine (53.64%), alanine (46.17%), and isoleucine (44.73%) levels. LPP had high phenolic content (25.10 mg/g GAE) and the changes in the antioxidant activities were not significant (*p* > .05). Moreover, chocolate production with LPP and control powder was also carried out under laboratory conditions. Hardness (1732.52 g), moisture content (0.60%), and water activity (0.37) of chocolate samples produced with low‐protein cocoa powder (LPC) were lower than those of the control sample. The Casson model well fitted to the rheological data (*R* ^2^ > .990) and chocolate samples showed elastic behavior. The removal of proteins from the cocoa was verified with Fourier transform infrared spectroscopy analyses. The melting temperatures of chocolates (31.84 and 31.54°C for control and LPC samples, respectively) did not change with the applied process. As a conclusion, it was revealed that the production of low‐protein cocoa powder and chocolate is feasible for patients with amino acid disorders with this study.

## Graphical abstract

The aim of the study was to produce low‐protein cocoa powder with enzymatic hydrolysis and precipitation method for patients with amino acid disorders. Significant reductions were obtained in the amino acid profile and protein content decreased by almost 40%. Chocolate production has also been carried out successfully and the quality parameters and the structural variations of chocolate samples were also determined.

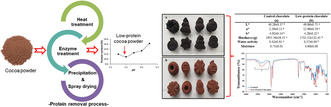

## 1 INTRODUCTION

Inherited metabolic diseases consist of 900 rare disorders, and about 30 of them can be treated with low‐protein diets. Phenylketonuria (PKU) is the most frequent one, which has a worldwide average prevalence of 1:10,000 newborns (Blau et al., 2010; van Spronsen et al., 2021). The other protein‐related disorders include maple syrup urine disease, homocystinuria, type I and II tyrosinemia, organic acidemias, and urea cycle disorders; however, their prevalence is lower than PKU (Blau et al., 2010; Mlčoch et al., 2018). The common aspect of these diseases is a deficiency or absence of a single enzyme causing a block in the metabolic pathway of an amino acid. Thus, the patients must follow a lifelong diet; otherwise, mental retardation, autism, motor development disorders, and other health complications are inevitable (Zannini et al., 2013). Although there are studies and applications related to gene therapy, enzyme therapy, or liver transplantation for treating amino acid disorder patients, they have some drawbacks. Enzyme therapy is a high‐cost treatment and can cause significant side effects. Gene therapy has been effective in mice, but it is still far from clinical trials. Therefore, dietary management remains the key therapy to keep blood amino acid concentrations within limits (Lichter‐Konecki & Vockley, 2019; van Spronsen et al., 2021).

A lifelong low‐protein diet policy has been accepted and implemented in recent years. Consumption of low‐protein food, a precursor‐free protein substitute with all other amino acids, and restriction of protein intake are the three main aspects of low‐protein diets (MacDonald & D'Cunha, 2007; van Spronsen et al., 2021). Meat and meat products, seafood, milk and dairy products, bread, nuts, and eggs are examples of protein‐rich foods that patients should avoid. Although some foods and beverages contain all nutritional supplements, except precursor protein, such as bars and powders; diverse food opportunities for the consumers are essential. For this, some foods can be modified to decrease their protein content by hydrolyzing proteins or using protein substitutes (Soltanizadeh & Mirmoghtadaie, 2014). These products can satisfactorily replace conventional ones that patients should not consume or are allowed to consume only a minimal amount in their daily diet. Studies have shown that these hydrolysates are produced from milk, whey protein, rice, and wheat flour. In the production of hydrolysates, the solubility has been increased by enzyme application, and protein has been removed by using different technologies, such as precipitation, adsorption, or ultrafiltration (Bu et al., 2020; Kılıç Büyükkurt et al., 2018; Silvestre et al., 2009; Soltanizadeh & Mirmoghtadaie, 2014). However, as far as authors' knowledge, there has been no study on the production of low‐protein cocoa powder with hydrolysis and precipitation.

It is essential to increase the variability of low‐protein foods and to buy these products from the market shelves and to reach them easily. These can make the patients' lives easier, just like diabetes and celiac patients. Therefore, this study aimed to investigate whether cocoa, a popular food with high protein content, can be turned into one of the products that amino acid disorder patients can consume. For this purpose, cocoa proteins were hydrolyzed and then decreased in protein level by isoelectric precipitation. After spray drying the cocoa solution, the prepared low‐protein cocoa powder was used for chocolate production. Solubility, sugar content, protein content, total phenolic content (TPC), antioxidant activities, and amino acid profile of cocoa powders were investigated. Furthermore, chocolate was produced with modified powder. Some quality parameters (color, texture, rheological properties, dynamic rheological behavior, moisture content, water activity, melting profile) and the structural variations (Fourier transform infrared spectroscopy) of chocolate samples produced with control and low‐protein cocoa powder (LPC) were determined.

## 2 MATERIALS AND METHODS

### 2.1 Materials

Methanol, iron (II) sulfate heptahydrate, sodium carbonate, Folin–Ciocalteu phenol reagent, gallic acid (≥98.0%) were supplied from Merck (Darmstadt, Germany). (±)‐6‐hydroxy‐2,5,7,8‐tetramethylchroman‐2‐carboxylic acid (Trolox, 97%), 2,2‐diphenyl‐1‐picrylhydrazyl (DPPH), TPTZ (2,4,6‐tripyridyl‐s‐triazine), and iron (III) chloride hexahydrate were purchased from Sigma (St. Louis, MO, USA). Cocoa powder was supplied from Barry Callebaut (Eskişehir, Turkey). Enzymes used in this study, Amylase (EC 3.2.1.1) AG 300 L (300 AGU/mL), Viscozyme L consisting of beta‐glucanase (EC 3.2.1.6), pectinase (EC 3.2.1.15), hemicellulase (3.1.1.73) and xylanase (EC 3.2.1.8) (≥100 FBGU/g), Alcalase (EC 3.3.21.62) (2.4 AU/g) were provided from Novozymes Enzim Dis Tic. Ltd. Sti (Istanbul, Turkey).

### 2.2 Low‐protein cocoa powder production

**FIGURE 1:**
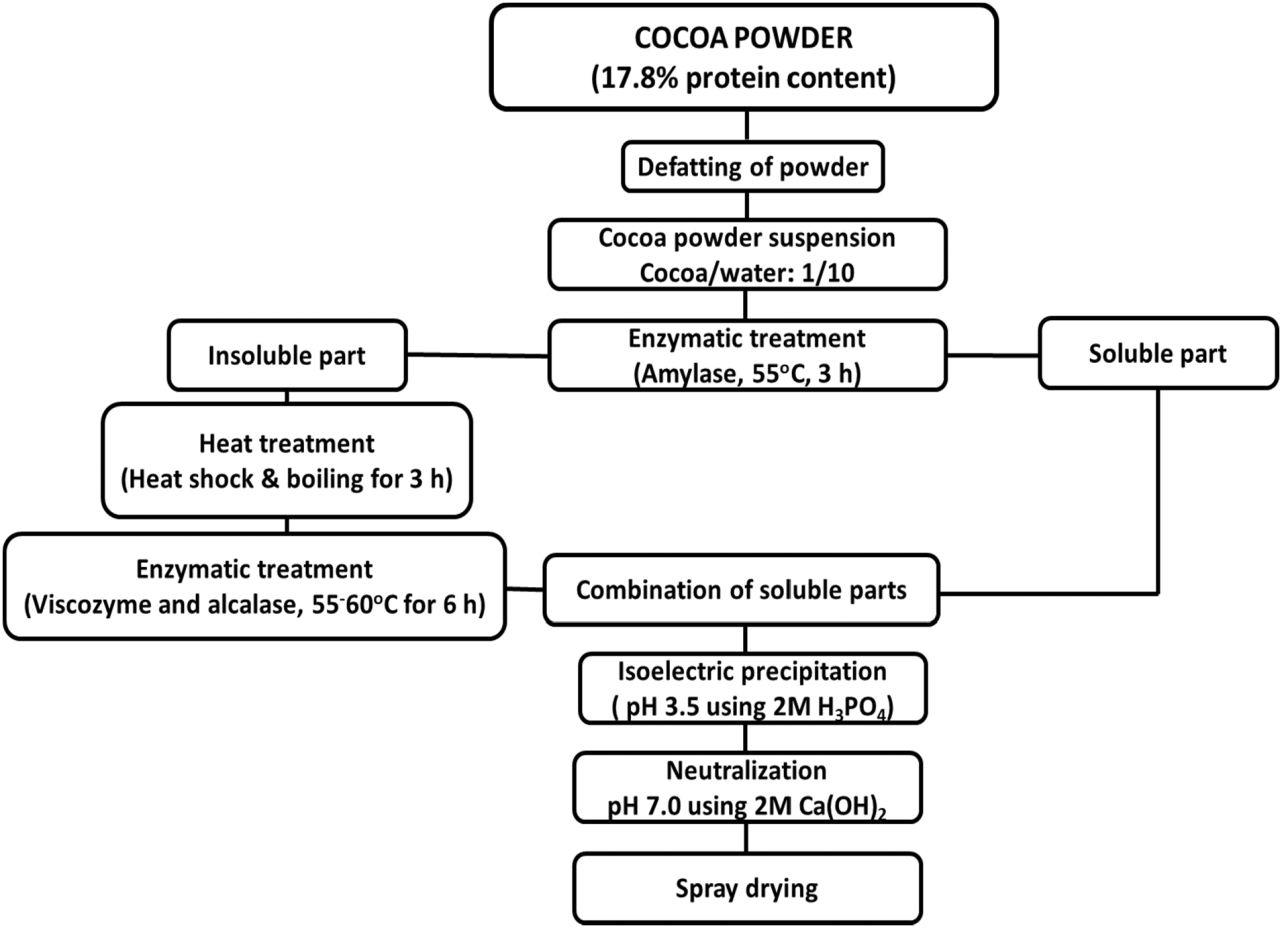
Production process of low‐protein cocoa powder.

Production steps of low‐protein content cocoa powder (LPP) are shown in Figure 1. Cocoa powder was first defatted three times using hexane and dried in an oven at 30°C for 24 h. Defatted cocoa powder was suspended in distilled water at 10 g/100 mL and heated on a magnetic stirrer at 55°C for 1 h. Amylase was added at a ratio of 1:10 (w/w, enzyme: substrate ratio), and the reaction was allowed for 3 h under recommended conditions (at pH 6 and 55°C). Amylase AG 300 L enzyme is a fungal glucoamylase produced from a selected strain of *Aspergillus niger*. It is a starch degrading enzyme that catalyzes the hydrolysis of 1.4‐alpha bonds in starch and also 1.6‐alpha bonds. The obtained suspension was centrifuged at 13,130 × *g* for 15 min to separate the soluble part and kept at 4°C. The insoluble part of the suspension was frozen to −18°C and followed by shock heating with distilled water at a temperature of between 95 and 100°C. It was allowed to continue until the temperature had stabilized (approximately 30 min) to obtain a further treated suspension. Then, it was boiled under the condenser for 3 h to loosen the tight structure of the cell walls. After heat treatment, Viscozyme, which provided tissue hydrolysis and consequent release of proteins from plant material, was applied at a ratio of 1:10 for 3 h (at pH 6 and 60°C). It is a cell wall degrading enzyme complex consisting of beta‐glucanases, pectinases, hemicellulases, and xylanases (Bu et al., 2020). To further break down the proteins, pH and temperature were adjusted to 7 and 50°C, and Alcalase was added for more 3 h. The enzymatic treatments were terminated by raising the temperature to 95°C for 5 min, and the hydrolysates were centrifuged at 13,130 × *g* for 15 min to discard the precipitates. The soluble parts were combined, and isoelectric precipitation was applied at pH 3.5 using 2 M H_3_PO_4_ solution. After centrifugation, the pH was increased to 7.0 using 2 M Ca(OH)_2_ solution. The pH was adjusted using Ca(OH)_2_ and H_3_PO_4_ instead of NaOH and HCl to avoid taste change by means of salt formation. Ca(OH)_2_ and H_3_PO_4_ formed calcium phosphate, a highly insoluble salt, and was easily separated from the soluble part (Blondeel et al., 2009; Bu et al., 2020). The precipitates were again discarded with centrifugation, and finally, the solution was spray‐dried.

Maltodextrin was used for spray drying of cocoa powder. The feed solution was prepared in a ratio of 1:1 w/w (maltodextrin: the dry matter of cocoa solution). The spray drying was performed on a Mini Spray Dryer Büchi B‐290 (Büchi Labortechnik AG, Flawil, Switzerland). A spray nozzle 0.7 mm internal diameter and compressed air at 6 bar were employed. The volumetric feed flow and air flow for atomization were programmed to 8.0 mL/min and 480 L/h, respectively. The set temperature was 160°C. The proportions of maltodextrin to cocoa solution and other conditions were determined with preliminary experiments.

### 2.3 Cocoa analysis

#### 2.3.1 Determination of solubility

To determine the solubility of cocoa powders, 10 g of sample was mixed with 90 g of distilled water and agitated at 20°C for 1 h. Then, the mixture was poured in a tube and centrifuged at 13,130 × *g* for 5 min. The upper soluble layer was used to measure the dry matter content (Blondeel et al., 2009).

#### 2.3.2 Determination of soluble sugar content

Glucose and fructose concentrations of samples were determined according to the method of Cerit and Demirkol (2021). Obtained cocoa solution before spray drying process was filtered with the 0.45 μm nylon filters. Ten microliters of the sample was injected to the HPLC system (Hitachi, Tokyo, Japan), which consisted of L‐2130 pump, L‐2200 autosampler, L‐2490 refractive index detector, L‐2300 oven. Isocratic elution was performed with an acetonitrile: water (85:15) solution at a flow rate of 0.8 mL/min. Column (GL Sciences, Inertsil NH_2_ column, 250 mm × 4.6 mm i.d.) operating temperature and injection volume were 40°C and 10 μL, respectively. Sugar concentrations were calculated with calibration curves prepared in the range of 0–5000 ppm. The *R* ^2^ values of glucose and fructose were .998 and .999, respectively.

#### 2.3.3 Protein analysis

The protein contents of samples were determined using the Kjeldahl method. Protein nitrogen was converted to % protein using the coefficient 6.25 (AOAC, 2000). Protein content was determined for each pH case (pH: 3.0. 3.5. 4.0. 4.5. 5.0).

#### 2.3.4 Amino acid profile with LC–MS/MS

Amino acid profile of samples was performed with the LC–MS/MS system. The procedure was performed by the JASEM's protocol (Table 1). For the quantification of amino acids (L‐taurine, L‐phenylalanine, L‐tyrosine, L‐leucine, L‐isoleucine, L‐methionine, L‐valine, L‐glutamic acid, L‐aspartic acid, L‐threonine, L‐serine, L‐alanine, L‐glycine, L‐proline, L‐cystine, L‐arginine, L‐ornithine, L‐histidine, L‐lysine), first, 0.5 g of the sample was taken into a screw‐capped glass tube and 4 mL of the JASEM Amino Acid Reagent (JASEM JSM‐CL‐508, Istanbul, Turkey) were added for hydrolyses reaction at 110°C for 24 h. Then, the hydrolysate was centrifuged at 5000 × *g* for 5 min, and the supernatant was diluted to 1 mL with distilled water. This procedure was repeated to obtain 800‐fold dilution. Diluted hydrolysate (50 μL) was transferred to a vial, and 50 μL of stable isotope labeled internal standard and JASEM acidic hydrolysis reagent were added. The JASEM quantitative amino acids kit protocol (Sem Laboratuvar Cihazları A. Ş, Istanbul, Turkey) were applied for LC–MS/MS analysis. The analysis was carried out on an Agilent 1260 Infinity HPLC system (Agilent Technologies, Santa Clara, USA) connected to an Agilent 6460 tandem mass spectrometer equipped with electro spray ionization (ESI) probe. Three milliliters of aliquot were injected into Jasem amino acid column (JASEM JSM‐CL‐575) with a flow rate of 0.7 mL/min. The results were expressed as g amino acid/kg sample (Bilgin et al., 2019; Tabak et al., 2021).

**TABLE 1:** LC–MS/MS analytical parameters.

Initial pressure	110 bar
Column temperature	30°C
Polarity	Positive
Gas temperature	150°C
Gas flow	10 L/min
Sheath gas temperature	400°C
Sheath gas flow	10 L/min
Nebulizer pressure	40 psi
Capillary voltage	2000 V (positive)

#### 2.3.5 Preparation of the extracts for the determination of phenolic content, antioxidant activities

The samples were defatted at the beginning of the process. Therefore, the extraction was directly applied according to Capanoglu et al. (2008) method with some modifications. A hundred milligram of cocoa powder was weighed and 3 mL of methanol:water (75:25, v/v) solution was added. The solutions were vortexed and they were incubated in the ultrasonic bath (Bandelin Sonorex, RK 100H, Berlin, Germany) for 15 min to increase the extraction efficiency. After centrifugation (13,130 × *g*, 4°C, 10 min), supernatants were separated and the above procedure was repeated twice. All supernatants were collected, and the final volume was adjusted to 10 mL.

#### 2.3.6 Determination of total phenolic content and antioxidant activities

The prepared extracts were diluted to one‐fifth ratio for TPC analysis. Then, 2 mL of distilled water and 0.2 mL of Folin–Ciocalteu reagent were added, and kept for 3 min. After the addition of 1 mL of sodium carbonate (20% w/v) solution, the mixture was incubated for 1 h at room temperature. The absorbance was recorded at 765 nm wavelength by using a UV–VIS spectrophotometer (Shimadzu UV mini 1240, Kyoto, Japan), and the standard curve was created with 0–500 ppm gallic acid solutions (*R* ^2^ = .998) (Wojdyło et al., 2007).

The antioxidant activities of cocoa powders were performed using two spectrophotometric methods: DPPH scavenging activity and FRAP assay. DPPH scavenging activity was determined according to Brand‐Williams et al. (1995) with some modifications. Briefly, 200 μL of prepared extracts were diluted in a ratio of 1:10 and mixed with 3 mL of 0.051 mmol/L DPPH solution, followed by incubation at room temperature for 30 min. The absorbance was read at 517 nm wavelength, and different concentrations of standard Trolox solution (0–50 ppm) were prepared to construct the standard curve (*R* ^2^ = .996).

FRAP assay was performed using the method developed by Benzie and Strain (1996). In a test tube, 0.1 mL of extract (dilution factor. 1:10), 8 mL of FRAP reagent prepared by mixing 300 mmol/L acetate buffer (pH 3.6), 10 mmol/L TPTZ, 20 mmol/L FeCl_3_.6H_2_O at the ratio of 10:1:1 (v/v), and 1.2 mL of distilled water were added. The mixtures were incubated in a 37°C water bath for 15 min. The absorbance was determined at 593 nm, and the calibration curve was constructed with standard FeSO_4_ solutions (0–1000 ppm. *R* ^2^ = .999).

### 2.4 Chocolate production

The ingredients used for chocolate production were cocoa, sugar and cocoa butter in a ratio of 14%, 46% and 40%, respectively. Other ingredients such as milk fat, or milk powder were not added because this product was prepared for PKU patients. Codex Alimentarius (2003) indicates that the chocolate shall not contain less than 14% fat‐free cocoa solid; therefore, the recipe of chocolate was adjusted according to it. First, cocoa butter was liquefied by the bain‐marie method, and then, the cocoa powder was added and mixed with a mixer (Fakir‐Mezza plus, Vaihingen, Germany). The mixture was ground in melanger (Santha, Utah, USA). Lecithin was added after 6 h of thinning process and mixed for more 30 min. Finally, the chocolate mass was cooled to 28°C, then heated to 32°C and tempering was completed (Cerit et al., 2016). Chocolate production was repeated for unprocessed cocoa powder (control sample).

### 2.5 Chocolate analysis

#### 2.5.1 Color analysis

The color of the chocolates was determined with a Colorimeter PCE‐CSM 7 (PCE Instruments, Southampton, UK). The color parameters of lightness (L*), redness (a*), and yellowness (b*) were recorded. The device was calibrated prior to measurements.

#### 2.5.2 Texture analysis

The texture of chocolate samples was measured using a texture analyzer (CT‐3, Brookfield Engineering Laboratories, Inc., Middleboro, USA). The thickness of the chocolate sample was 3 mm and a flat‐ended probe was used to determine hardness (TA7, Brookfield Engineering Laboratories). Three measurements were conducted for each sample by the conditions of 0.5 g trigger load and 1 mm/min head speed.

#### 2.5.3 Rheological properties of chocolates

Rheological analysis was performed using a Malvern Kinexus Pro Rheometer (Worcestershire, UK) equipped with parallel plate geometry under controlled temperature. The diameter of the plates was 40 mm and the gap between the plates was set to 1 mm. Shear rate values were between 0.1 and 100 s^−1^. The chocolates were melted in a water bath to adjust the temperature to 40°C. Tests were also conducted at 40°C. The rheological data were fitted to the Casson model as described in Equation (1);

(1) $$\sqrt{\sigma }=\sqrt{a}+\sqrt{b}\sqrt{\gamma }$$

where *σ* is shear stress (Pa), *a* is yield stress (Pa), *b* is Casson viscosity (Pa.s), and *γ* is shear rate (1/s).

#### 2.5.4 Dynamic rheological behavior of chocolates

Dynamic rheological behaviors of chocolate samples were investigated with strain sweep and frequency sweep tests. Strain sweep test was performed at 10 rad/s constant frequency and 0.001%–100% strain. Storage module (G′) and loss module (G″) values were recorded in the frequency range of 0.1–100 rad/s with 0.002% strain at 40°C. In all cases, the temperature of the samples was kept at 40°C.

#### 2.5.5 Moisture content analysis

The moisture content of chocolate samples was analyzed with AND MX‐50 (Tokyo, Japan) moisture analyzer (Asghar et al., 2017). Chocolate samples were cut into small pieces and particles were placed in the aluminum boat. The temperature of the device was set to 105°C and moisture percentage was recorded at the end of the analysis.

#### 2.5.6 Water activity analysis

The water activity of chocolate samples was measured using an AquaLab® water activity meter (Decagon Devices Inc., Pullman, WA, USA). Results were reported as the average of three replicates.

#### 2.5.7 Fourier transform infrared spectroscopy analysis

Fourier transform infrared spectroscopy analyses of the chocolate samples produced with control and low‐protein cocoa powder were performed on a Perkin Elmer spectrometer (Massachusetts, USA) using the Spectrum Two Model by ATR technique. The spectra were obtained at room temperature in the range of 4000 and 400 cm^−1^ and data were analyzed with Spectrum Software.

#### 2.5.8 Differential scanning calorimetry analysis

Melting behavior of the chocolate samples was determined with differential scanning calorimetry analysis (Seiko, DSC 7020, Hitachi, Japan) (Toker et al., 2018). Samples were placed into aluminum pans and were hermetically sealed. Then, the samples were heated from −80 to 80°C at 10°C/min in N_2_ stream.

### 2.6 Statistical analysis

The results are expressed as mean ± standard deviation. The statistical analyses were performed using SPSS (version 11.5; SPSS Inc., USA). *T*‐test (*α* = .05) was applied to show the difference between samples.

## 3 RESULTS AND DISCUSSION

### 3.1 Cocoa properties

Most of the foods for amino acid metabolic disorder patients are in the form of mixtures of specific and limited nutritional components. These patients can not generally consume basic food sources in their diets (van Spronsen et al., 2021). As far as we know, there is no research about the production of low‐protein cocoa powder. Cocoa is a popular food with a characteristic taste and unique properties such as health‐promoting effects. It is a primary raw material of chocolate and is also used as an ingredient in many preparations, such as cookies, drinks, bread, and cakes (Rucker, 2009; Yiğit et al., 2021). For the reasons mentioned above, in this study, low‐protein cocoa powder (LPP) was produced to provide an important alternative and diversity in patients' diets and improve their life quality.

Cocoa powder has low solubility due to the high fat content and a large number of polysaccharide‐based carbonyl groups (Omobuwajo et al., 2000). Therefore, the first step of low‐protein cocoa production was increasing the solubility of powder with heat and enzymatic treatments. Amylase and Viscozyme were used for starch and cell wall degradation, respectively. Alcalase was also added for further breakdown of the proteins in cocoa powder. The reducing sugar contents of cocoa powder before and after treatment are given in Table 2. Although reducing sugars were not detected with the HPLC‐RID detector in the control powder (sugar content is specified as 0.4 g/100 g on the product label), the amount of glucose and fructose were found to be 21.34 g and 1.14 g per 100 g of LPP. It has been reported that free sugar content of cocoa bean is not more than 2%–4% (dry weight) and predominant sugars are sucrose, fructose, and glucose. While reducing sugar increases during fermentation due to enzymatic reactions, it decreases after drying and roasting processes. Therefore, reducing sugar was not detected in control sample; however, it was determined after enzymatic treatments. Moreover, chemical conversion of sucrose into glucose and fructose due to pH drop during processing may also increase the reducing sugar concentration (Afoakwa et al., 2013; Redgwell et al., 2003). The solubility of cocoa also increased from 28.61% to 50.69% (Table 2). Blondeel et al. (2009) developed a method for producing a soluble cocoa product from cocoa powder. Their invention provided solubility of cocoa in water of at least 50%. It was reported that 14.5% of starch and 22.5% of cellulose converted to glucose, dextrin, and glucose oligomers which explains the increase in solubility in this study (Blondeel et al., 2009).

**TABLE 2:** Sugar content, solubility, and antioxidant activity of cocoa powders.

	Control cocoa powder	Low‐protein cocoa powder
Glucose (g/100 g)	nd	21.34 ± 1.21
Fructose (g/100 g)	nd	1.14 ± 0.30
Solubility (%)	28.61 ± 0.08^b^	50.69 ± 0.07^a^
TPC (mg GAE/g)	15.82 ± 0.76^b^	25.10 ± 1.44^a^
DPPH (mg Trolox/g)	22.37 ± 0.25	22.70 ± 0.17
FRAP (mg FeSO_4_/g)	222.14 ± 1.88	224.12 ± 0.77

*Note*: Different letters within the same row indicate statistically significant differences (*p* < .05).

Abbreviation: nd, not detected.

**FIGURE 2:**
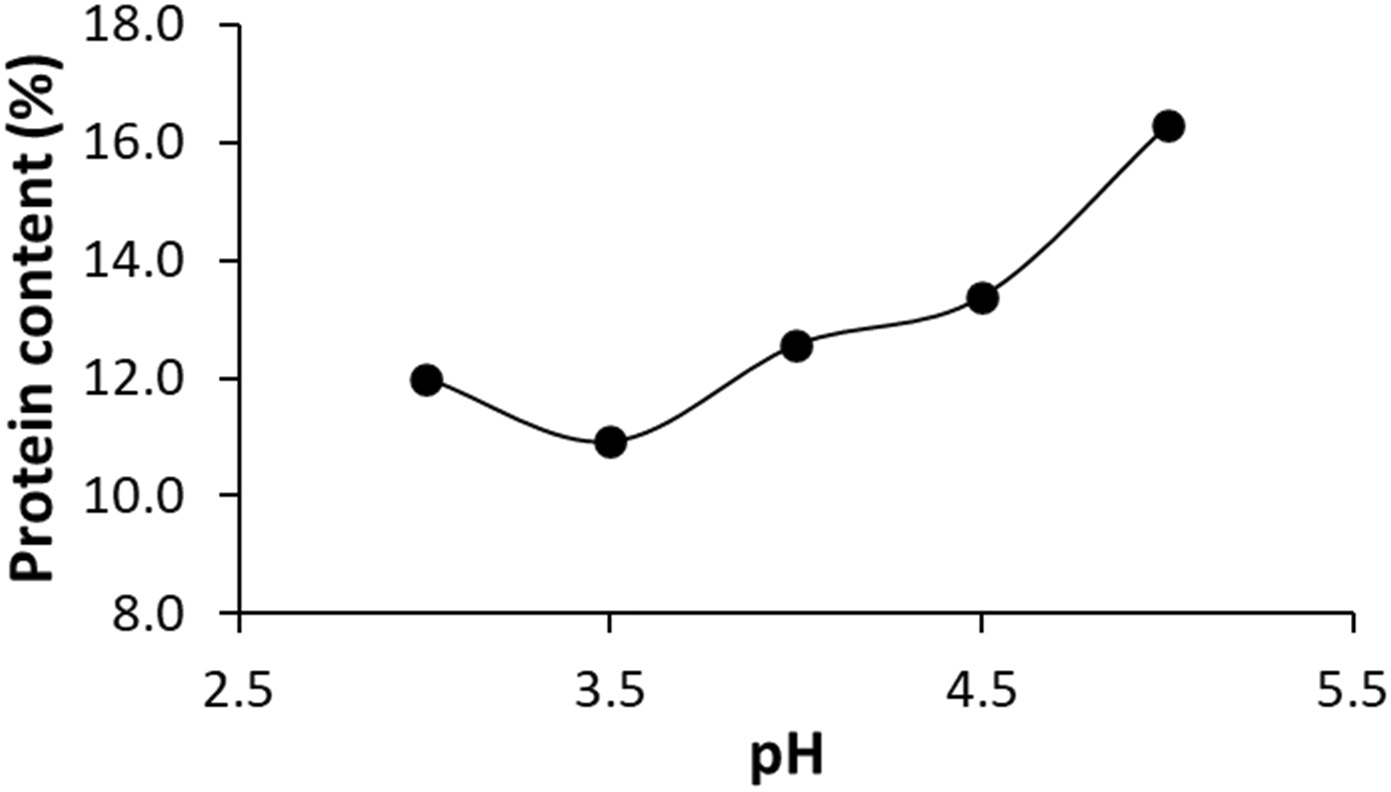
Protein content of cocoa powder at different pH values.

The protein content of cocoa powder was reported as approximately 18–19 g/100 g in the literature (Rucker, 2009) and it was determined as 17.8 g/100 g in this study. Preliminary studies showed that precipitation of proteins was highest when the pH of the solution was adjusted to 3.5. Therefore, proteins were precipitated with H_3_PO_4_ solution at pH 3.5 (Figure 2). Then, the cocoa solution was spray‐dried with the help of maltodextrin, and the amino acid profile of both control and LPP was quantified with the LC‐MS/MS system (Table 3). Glutamic acid (42.78 mg/kg) was the most relevant amino acid among the sample. Serine occupied the second position in control samples with a value of 21.27 g/kg. Cystine, glycine, and taurine were not detected. Adeyeye et al. (2010) reported that glutamic acid and cystine were the most and least abundant amino acids in cocoa nibs, respectively, similar to our findings.

**TABLE 3:** Content and type of amino acid as quantified by LC–MS/MS.

Amino acid	Control cocoa powder (g/kg)	Low‐protein cocoa powder (g/kg)	Reduction (%)
Alanine	3.69 ± 0.01^a^	1.98 ± 0.02^b^	46.17 ± 0.03
Arginine	11.58 ± 0.01^a^	7.08 ± 0.03^b^	38.85 ± 0.04
Aspartic acid	19.00 ± 0.02^a^	11.34 ± 0.04^b^	40.34 ± 0.09
Cystine	nd	nd	nd
Glutamic acid	42.78 ± 0.02^a^	28.64 ± 0.08^b^	33.06 ± 0.01
Glycine	nd	nd	nd
Histidine	5.46 ± 0.01	5.71 ± 0.01	0.00 ± 0.00
Isoleucine	1.53 ± 0.17^a^	0.85 ± 0.01^b^	44.73 ± 0.44
Leucine	10.54 ± 0.92^a^	4.89 ± 0.05^b^	53.64 ± 4.54
Lysine	2.36 ± 0.09^a^	0.62 ± 0.02^b^	73.65 ± 0.19
Methionine	1.50 ± 0.01	nd	100.00 ± 0.00
Ornitine	12.68 ± 0.01^a^	11.35 ± 0.02^b^	10.49 ± 0.14
Phenylalanine	11.63 ± 0.07^a^	9.87 ± 0.15^b^	15.12 ± 0.17
Proline	18.91 ± 0.29^a^	15.47 ± 0.04^b^	18.18 ± 1.46
Serine	21.27 ± 0.02^a^	15.07 ± 0.05^b^	29.16 ± 0.29
Threonine	17.04 ± 0.04^a^	15.09 ± 0.01^b^	11.45 ± 0.17
Tyrosine	5.30 ± 0.47	5.19 ± 0.35	2.05 ± 0.01
Valine	14.28 ± 0.02^a^	13.07 ± 0.01^b^	8.48 ± 0.00
Taurine	nd	nd	nd

*Note*: Different letters within the same row indicate statistically significant differences (*p* < .05).

Abbreviation: nd, not detected.

When the amino acid profile of LPP was examined, significant reductions in amino acid concentrations were obtained except for histidine and tyrosine (*p* < .05). The most remarkable reduction rates in amino acid concentrations were 100%, 73.65%, 53.64%, 46.17%, and 44.73% for methionine, lysine, leucine, alanine, and isoleucine, respectively. Among these amino acids, reducing leucine and isoleucine level is important for MSUD patients who must follow lifelong dietary restrictions in terms of branched‐chain amino acids (Blackburn et al., 2017). Phenylalanine removal was 15.12% which is also significant for PKU patients (Lichter‐Konecki & Vockley, 2019). In OAs and UCD patients, reducing nitrogen load is the purpose of dietary treatment to prevent intoxication (Batshaw et al., 2014; Fujisawa et al., 2013). No reports were found in the literature concerning the removal of proteins from cocoa powder. However, data for some other foods have been reported about reduction of proteins using different post‐treatment steps. For instance, in the study of Su et al. (2021), removal of phenylalanine from egg white powder by enzymatic hydrolyses followed by activated carbon treatment was examined. They reported that the percentage of Phe removal was up to 97% which was higher than our study. The removal efficiency of different amino acids may be associated with various factors such as enzyme type, process conditions, or applied treatments (Su et al., 2021).

Epidemiological studies have shown that cocoa contains high concentration of polyphenols that is associated with positive effects on health, such as decreasing the risk of cardiovascular disease and amelioration of insulin sensitivity (Buijsse et al., 2010; Grassi et al., 2008). Therefore, changes in TPC and antioxidant activity of LPP were examined and the results are shown in Table 2. While TPC of the control sample was determined as 15.82 mg/g GAE, it was 25.10 mg/g GAE in LPP. Gültekin‐Özgüven et al. (2016) were reported the TPC of cocoa powder as 18.0 mg catechin equivalent/g in dry defatted base. In another study, TPC changed from 41.55 mg GAE/g in natural cocoa powder to 10.67 mg GAE/g in alkalized cocoa powder, which is in parallel with our findings (Todorovic et al., 2017). The TPC value of LPP was significantly higher compared to the control sample. It was thought that the reasons for this difference might be the heat treatment and applied enzymes during the process. Because the cellular structure degradation of cocoa occurred, bound phenolic compounds and peptides of smaller molecular weight were released as a consequence of both heat and enzymatic treatments. These peptides may also have increased the phenolic content of LPP sample due to their antioxidant properties (Sarmadi et al., 2011). In the control sample, phenolic compounds may be bound to proteins or cell wall polysaccharides, which block the extractability of phenolic compounds from the cocoa matrix (Oracz et al., 2015). In addition, the phenolic content was measured with the Folin–Ciocalteu reagent, which reacts with other reducing compounds such as pigments, carbohydrates, and Maillard reaction products. Because the reducing sugar content increased in LPP, the formation of melanoidins could provide the increase in the TPC (Arlorio et al., 2008; Coghe et al., 2006; Ioannone et al., 2015).

There is a growing interest in the content of antioxidant compounds in foods, and many studies have investigated cocoa's antioxidant properties that affect the consumption rate of the products. In this study, the antioxidant activities of cocoa samples were determined with two spectrophotometric methods. The results of DPPH scavenging activity and FRAP are given in Table 2. According to the results, DPPH values of control and low‐protein cocoa samples were 22.37 mg/g Trolox and 22.70 mg/g Trolox, respectively. FRAP values were also found to be 222.14 and 224.12 mg/g FeSO_4_, respectively. There were no significant differences between samples when antioxidant results were compared (*p* > .05). The fact that there was no change in the antioxidant activity as a result of the process is a positive outcome for this study.

### 3.2 Chocolate properties

**FIGURE 3:**
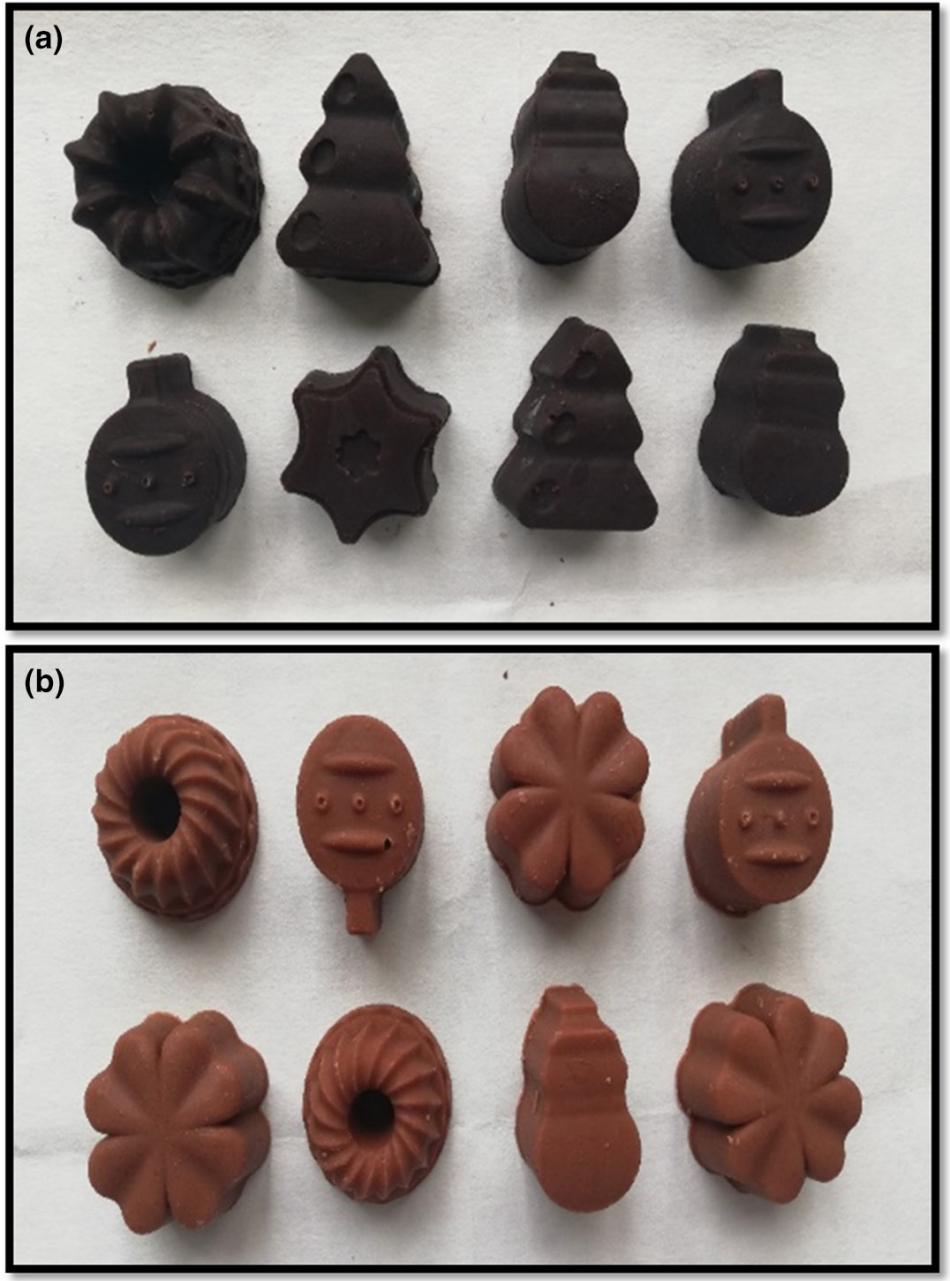
Chocolate samples produced with control cocoa powder (a) and low‐protein cocoa powder (b).

One of the most popular cocoa products is chocolate which consists of cocoa butter, sugar, and other flavoring agents such as vanillin, milk ingredients, or spices. In this study, cocoa, cocoa butter, and sugar were used to produce chocolate for patients with the amino acid disorder. The main aim was to see if the produced low‐protein cocoa could be used to make chocolate. The same procedure was applied during the production, including mixing the ingredients, thinning and tempering processes, respectively (Cerit et al., 2016). Chocolates produced with control and low‐protein cocoa under laboratory conditions are shown in Figure 3. The color of a food product is the primary quality parameter to attract the consumer. When the colors of chocolate samples were compared, significant difference was seen. Chocolate with low‐protein cocoa (LPC) seemed lighter and redder than the control sample. The difference in appearance was also proven by color values (Table 4). Although the L* value of the control chocolate was 40.28, it was 49.86 for LPC, which was significantly high. In addition, there was an increase in the surface redness (a*) and yellowness (b*) after maltodextrin addition. The main reason for this difference is the addition of maltodextrin during spray drying to increase efficiency. In preliminary studies, a maltodextrin concentration of 1:2 w/w (maltodextrin: the dry matter of cocoa solution) was tried. However, the high sugar amount present in the low‐protein cocoa strongly interacted with water molecules which caused agglomeration. In the study of Ribeiro et al. (2020), maltodextrin addition on the flow properties of cocoa pulp powder obtained by spray and freeze drying was investigated. They used 15% and 30% maltodextrin DE20 in cocoa pulp samples and reported that the higher the maltodextrin concentration in cocoa pulp, the lower the product agglomerated by spray drying (Ribeiro et al., 2020). Therefore, the maltodextrin concentration of 1:1 w/w was used during spray drying, which resulted in a lighter color.

**TABLE 4:** Physical properties of chocolate samples.

	Control chocolate	Low‐protein chocolate
L*	40.28 ± 0.37^b^	49.86 ± 0.75^a^
a*	2.29 ± 0.11^b^	12.90 ± 0.19^a^
b*	−4.92 ± 0.14^b^	4.28 ± 0.22^a^
Hardness (g)	1955.50 ± 56.53^a^	1732.52 ± 132.45^b^
Water activity	0.42 ± 0.01^a^	0.37 ± 0.00^b^
Moisture content (%)	0.71 ± 0.01	0.60 ± 0.00

*Note*: Different letters within the same row indicate statistically significant differences (*p* < .05).

The texture is one of the critical quality attributes of chocolate and Table 4 reveals the effect of maltodextrin addition on the hardness of samples. Hardness values were 1955.50 and 1732.52 g in control and LPC samples, respectively. Hardness can be defined as the force required to achieve a certain deformation and is an indicator of rigidity. It depends on a variety of factors, which are mainly lipid and dispersed phase, tempering, particle size distribution, and composition (Barišić et al., 2021). In this study, the hardness value decreased in the LPC sample due to the maltodextrin content. Farzanmehr and Abbasi (2009) added polydextrose, inulin, and maltodextrin to milk chocolate and they reported that chocolates with polydextrose and maltodextrin were softer than controls similar to this study. Lim et al. (2021) were also used different sucrose replacers (inulin, fructo‐oligosaccharide, trehalose, or maltodextrin) to produce sucrose‐free dark chocolates. It was found that all sucrose‐free samples were softer than control samples which was attributed to particle–particle interactions in the chocolate (Lim et al., 2021).

**FIGURE 4:**
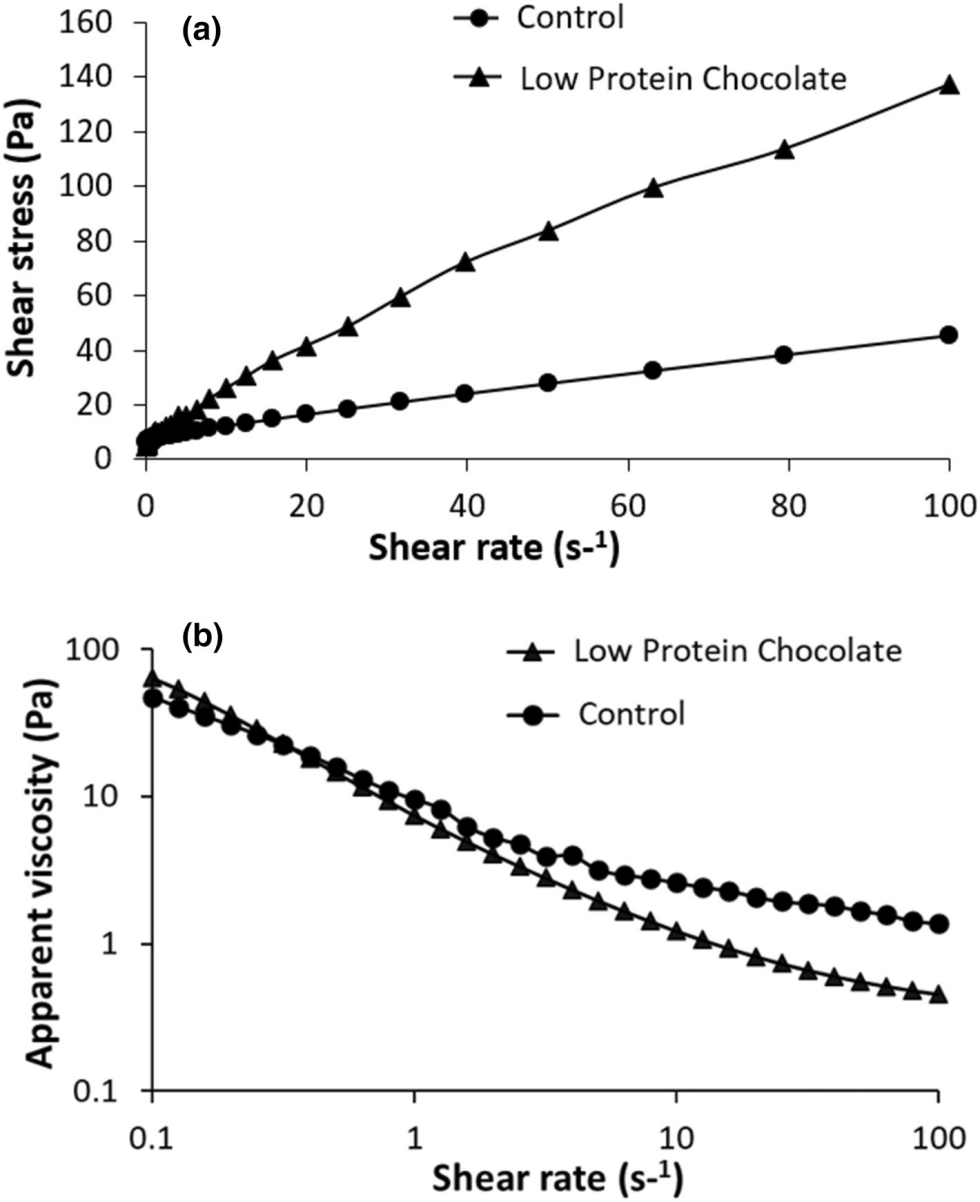
Flow behaviors of chocolates at 40°C: (a) shear stress versus shear rate; (b) viscosity versus shear rate.

The effect of using low‐protein cocoa powder on the flow behaviors of chocolates was investigated using steady shear measurements. The shear stress versus shear rate and apparent viscosity versus shear rate of chocolate samples are given in Figure 4a,b, respectively. The slope of shear stress versus shear rate was not constant, showing that the chocolates behaved as non‐Newtonian fluids. Moreover, viscosity decreased with increasing shear rate for both samples. This proved that the samples became shear‐thinning as expected and has been indicated by others (Kiumarsi et al., 2017; Vásquez et al., 2019). Experimental data of samples were well fitted to the Casson model, which has often been used to analyze the rheological properties of chocolates (Gonçalves & Lannes, 2010). *R* ^2^ values were .991 and .998 for control and LPC samples, respectively. Casson viscosity and Casson yield stress were obtained from the slope and intercept of this plot (Table 5). There was a significant difference between the viscosity values of chocolate samples (0.17 Pa.s for control and 0.98 Pa.s for LPC). It may be attributed to the polysaccharide structure of added maltodextrin, which increased the viscosity of low‐protein chocolate. An increase when replacing sucrose with maltodextrin was also observed in the study of Farzanmehr and Abbasi (2009). The Casson yield stress value was higher in the control sample (5.38 Pa), indicating that the amount of energy required to start flow was lower in the LPC sample (3.96 Pa). Such difference can be related to the plasticizing effect of bulking agents (such as maltodextrin) in the formulations (Cikrikci et al., 2017).

**TABLE 5:** The rheological parameters of the chocolate samples.

	Control	Low‐protein chocolate
Casson yield stress (Pa)	5.38 ± 0.37^a^	3.96 ± 0.05^b^
Casson viscosity (Pa.s)	0.17 ± 0.01^b^	0.98 ± 0.19^a^
*R* ^2^	.991	.998

*Note*: Different letters within the same row indicate statistically significant differences (*p* < .05).

**FIGURE 5:**
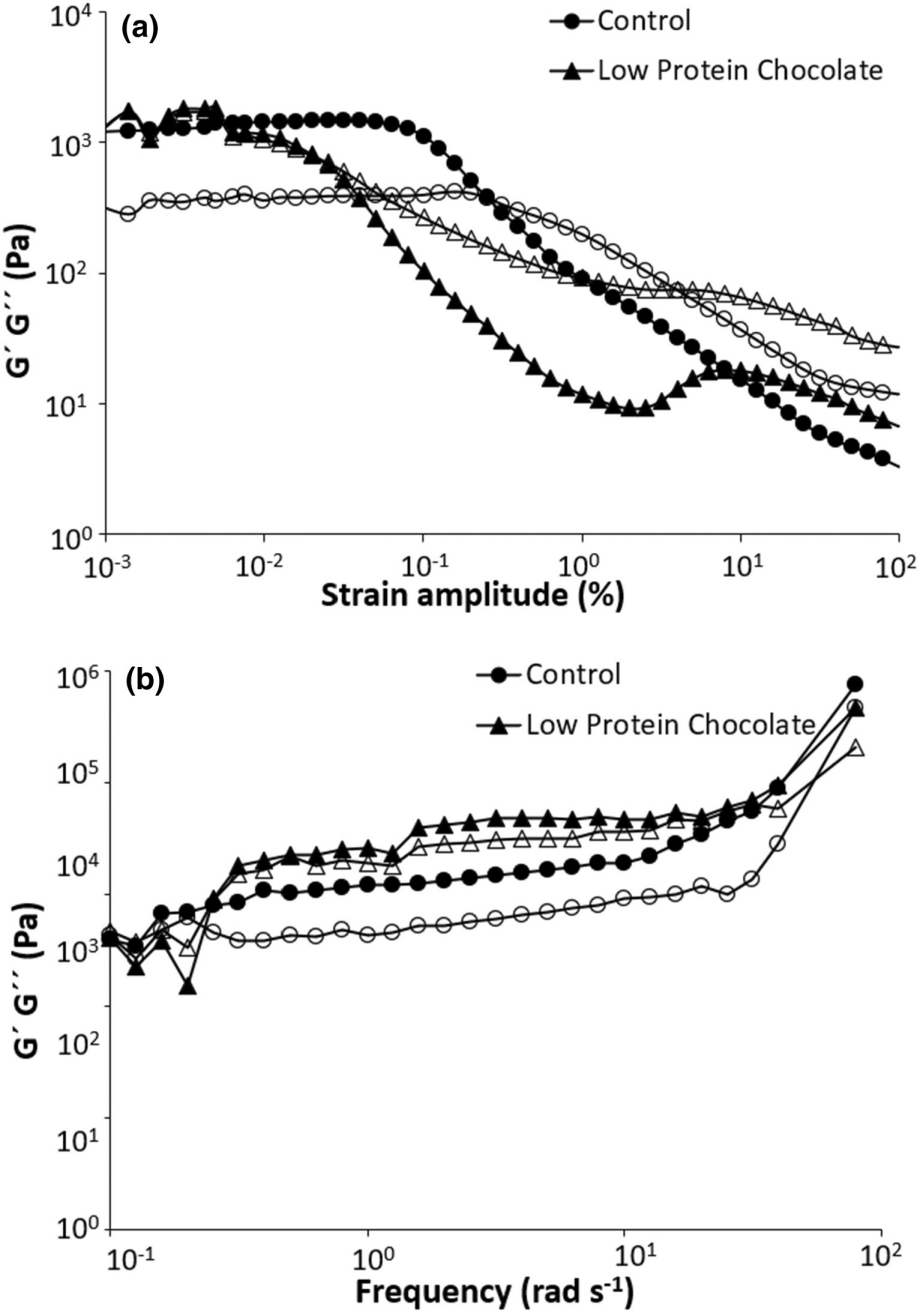
Strain sweep at frequency of 10 rad/s for chocolate samples (a) and frequency sweep for chocolate samples at deformation of 0.002% (b) (G′: filled symbols and G″: open symbols).

The linear viscoelastic region (LVR) was determined by performing strain sweep tests. Figure 5a shows strain sweeps for control and LPC samples at 40°C and a constant frequency of 10 rad/s. It can be observed that critical strain amplitude was about 0.08% for the control sample, while a narrower region was observed for LPC (approximately 0.002%–0.005%). Therefore, 0.002% strain was applied to assess the rheological properties of the chocolates. In the frequency sweep test, G′ and G″ refer to the index of a sample's elastic and viscous behaviors, respectively. As illustrated in Figure 5b, G′ values of both chocolate samples were higher than those recorded for G″ which shows the elastic behavior of the samples. It was reported that a high G′ value could be attributed to the formation of structural interactions between ingredients. Loghmani et al. (2022) examined the dynamic rheological properties of molten dark chocolates produced from sugar substitutes. They found that sucrose and sorbitol showed elastic behavior, while inulin and isomalt contained samples were mostly viscous (Loghmani et al., 2022). Similarly, Acan et al. (2021) used dried grape pomace as a sugar replacer in the chocolate spread formulation and reported higher G′ values than those recorded for G″ values.

Chocolate is a low water activity (*a*_w_) product and the experimental results show that the *a*_w_ values of both control (0.42) and LPC samples (0.37) were not beyond the upper limit (Table 4) (Chire‐Fajardo et al., 2017). While temperature and humidity during conching can affect the water activity of chocolate, the ingredients may also have a substantial effect. The most significant difference between the components of LPC and control samples was maltodextrin in this study. The LPC sample had lower water activity and moisture content than the control sample. The moisture content of regular chocolate is typically 0.5%–1.5% (Asghar et al., 2017). The findings show that the LPC sample may be less susceptible to microbial growth and more convenient for the shelf‐life.

**FIGURE 6:**
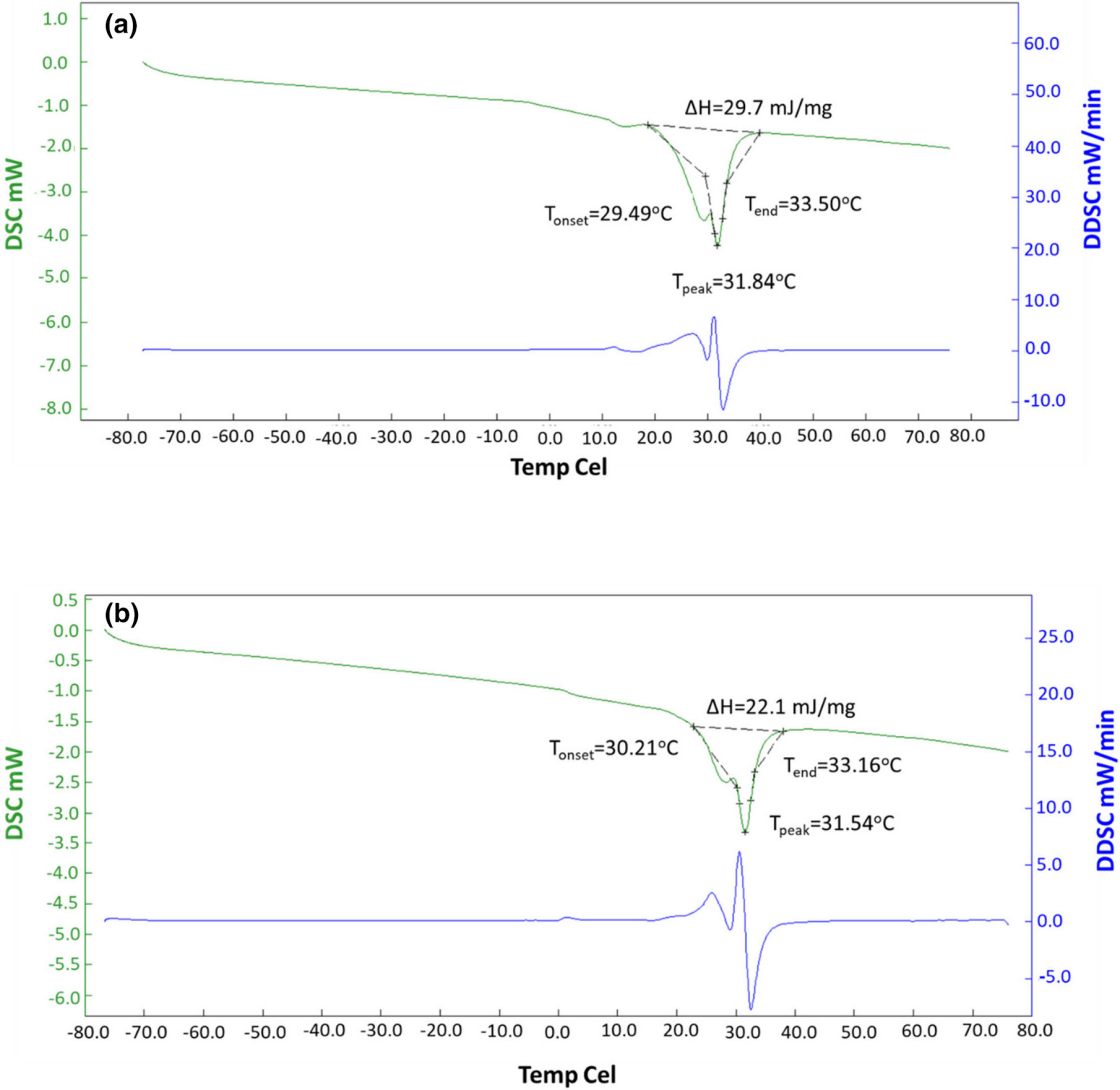
Differential scanning calorimetric measurements of chocolate samples produced with control cocoa powder (a) and low‐protein cocoa powder (b).

DSC is a common method used to determine chocolate's thermal properties, such as glass transition temperature, enthalpy values, and melting temperature. The advantages of DSC analysis are that it gives fast results and does not require extensive preprocessing (Ioannidi et al., 2021). In this study, the melting characteristics of chocolate samples were investigated and DSC curves are given in Figure 6. Onset temperature (*T*_onset_), peak temperature (*T*_peak_), end temperature (*T*_end_), and energy required for the complete melting of the samples (Δ*H*) were calculated using thermograms. *T*_onset_ is the temperature at which melting starts and it was found as 29.49 and 30.21°C for control and LPC samples, respectively. *T*_peak_ of control and LPC were recorded as 31.84 and 31.54°C, respectively. *T*_end_ represents the temperature at which complete liquefaction occurs (Toker et al., 2018). *T*_end_ values were also found as 33.50°C for the control sample and 33.16°C for the LPC sample. As can be seen, the temperatures showing thermal properties of chocolates were similar in both samples. The applied process did not affect the melting characteristics. However, Δ*H* values of control and LPC samples were 29.7 and 22.1 mJ/mg, respectively. The reason for the difference between Δ*H* values could be the presence of maltodextrin in LPC samples. In the study of Sarfarazi and Mohebbi (2020), sugar was replaced with inulin:maltodextrin mixture at different ratios and DSC analysis was applied. They reported that a higher Δ*H* value in the control sample (sugar added) than maltodextrin added sample (maltodextrin:inulin 75:25 added) could be associated with smaller particle size of the control sample causing higher particle–particle aggregation (Sarfarazi & Mohebbi, 2020).

**FIGURE 7:**
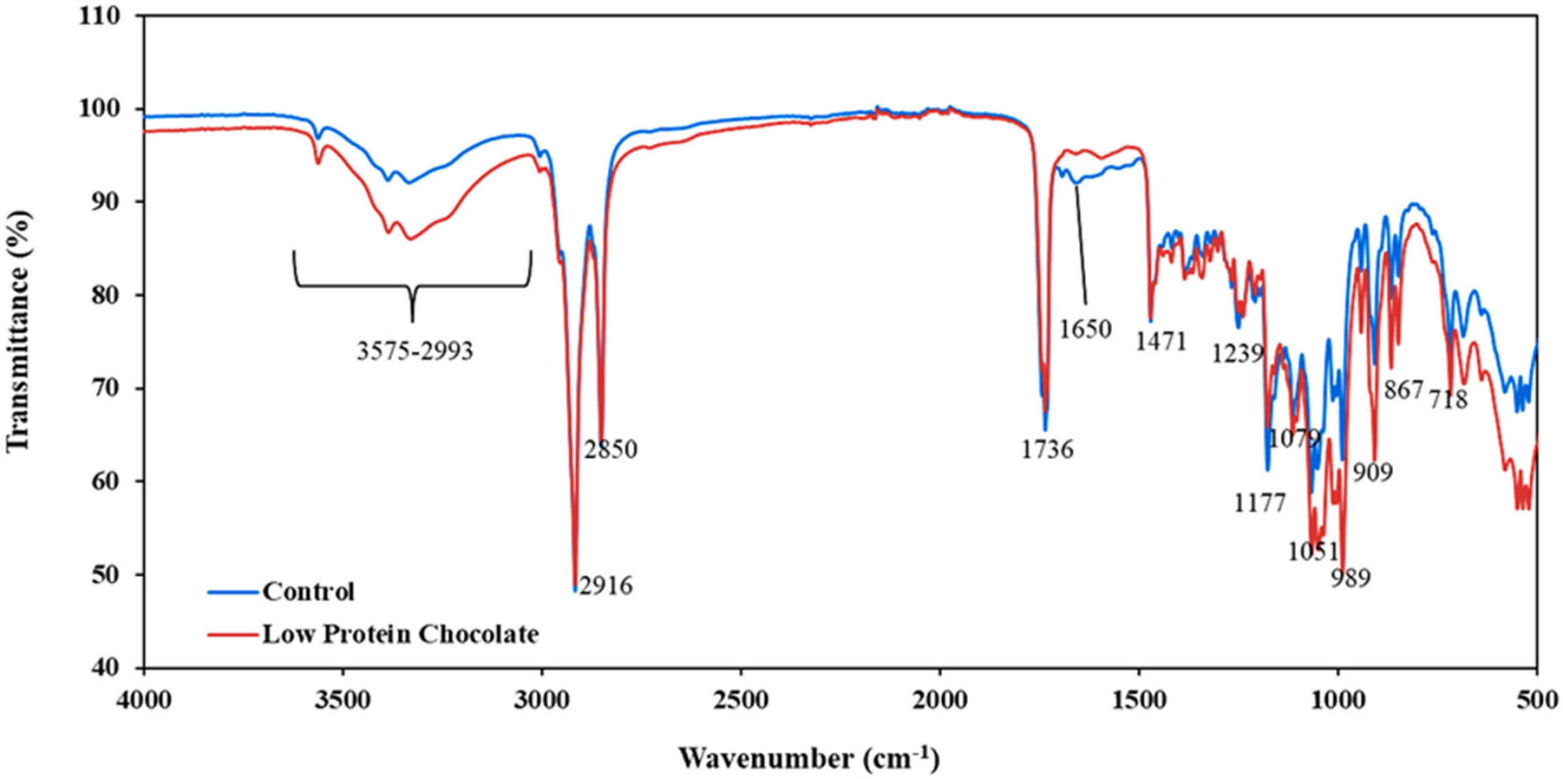
Fourier transform infrared (FTIR) spectra of chocolate samples.

Fourier transform infrared analysis was performed to reveal the differences between the functional groups in the chocolate samples (Figure 7). The peaks obtained with both control and LPC samples were almost identical, except for some differences between the transmittance values and the reduction of a few specific peaks. The most significant difference between the samples was observed at 1650 cm^−1^ wavenumber, which was related to the stretch of C=O bonds (amid I) in proteins. There was a peak at the control sample at this wavenumber while it reduced significantly in the LPC sample. Hence, it validated the efficiency of protein removal from the cocoa. The other main peaks were obtained at 3575–2993, 2916, 2850, and 1736 cm^−1^. The strong peaks at the 1500–500 cm^−1^ fingerprint region were mainly attributed to the carbohydrates (Cerit et al., 2023; Collazos‐Escobar et al., 2020; Vargas‐Muñoz & Kurozawa, 2020). The wide O‐H stretching vibration peaks were detected between 3575 and 2993 cm^−1^ corresponding to phenols and sugars. The transmission intensity of the LPC sample was lower than the control sample in this region due to the maltodextrin used in the spray drying process. The intense sharp overlapped peaks at 2916, 2850, and 1736 cm^−1^ were specific for lipids and attributed to cocoa butter present in the formulations. The first two peaks were due to the asymmetrical and symmetrical stretching vibration of C–H bonds in lipids (Deus et al., 2021; Grillo et al., 2019), and the last peak was assigned to and the last peak was assigned to stretching of C=O bonds adjacent to C‐O‐ ester groups (Craig et al., 2018).

## 4 CONCLUSIONS

In this study, low‐protein cocoa powder was produced with enzymatic hydrolysis and precipitation method. Although there have been limited studies on the producing low‐protein foods, this research is novel due to the product's originality. First, the solubility of cocoa was increased by heat and enzyme treatments, and then, the protein level was decreased by isoelectric precipitation. Then, the solution was dried with a spray dryer using maltodextrin as a carrier. Changes in solubility, sugar content, protein content, amino acid profile, and antioxidant activities were examined. The results indicated that the solubility increased significantly with the treatments, and protein content decreased by almost 40% according to the control sample. Moreover, significant reductions were obtained in the amino acid profile except for histidine and tyrosine. While the total phenolic content of low‐protein cocoa was higher, the antioxidant activities did not change. Finally, the obtained cocoa samples were successfully used for chocolate production. Low‐protein chocolate seemed lighter and redder than the control sample. Maltodextrin addition decreased the hardness, moisture content, and water activity. The Casson model provided the well fits with high *R* ^2^ values. Both control and low‐protein chocolate samples showed elastic behavior. DSC analysis showed that the melting temperature of the chocolate sample was not affected by applied process. FTIR analyses validated the removal of proteins from the cocoa. As a consequence, this study provides insight into production of alternative foods for amino acid disorder patients. Other than chocolate, low‐protein cocoa powder can also be utilized in other cocoa‐based products such as drinks, ice cream, or bakery foods. Therefore, the patients can reach a broad range of cocoa‐based products. In future studies, various methods such as ultrafiltration or activated carbon application can be tried to improve the efficiency of protein removal.

## AUTHOR CONTRIBUTIONS

**İnci Cerit:** Formal analysis (equal); methodology (equal); writing – original draft (equal); writing – review and editing (equal). **Könül Mehdizade:** Formal analysis (equal). **Ayşe Avcı:** Formal analysis (equal); writing – original draft (equal); writing – review and editing (equal). **Omca Demirkol:** Conceptualization (equal); methodology (equal); supervision (equal); writing – review and editing (equal).

## CONFLICT OF INTEREST STATEMENT

The authors declare no conflicts of interest.

## Data Availability

The data that support the findings of this study are available from the corresponding author upon reasonable request.
